# Overcoming Obstacles: Barriers to Virtual Care Use Among Video-Enabled Tablet Recipients in the Veterans Health Administration

**DOI:** 10.1007/s11606-023-08468-x

**Published:** 2023-11-01

**Authors:** Charlie M. Wray, Jacqueline M. Ferguson, Liberty Greene, Ashley Griffin, James Van Campen, Amy MJ O’Shea, Cindie Slightam, Donna M. Zulman

**Affiliations:** 1 Department of Medicine, University of California, San Francisco, San Francisco, CA USA; 2 Section of Hospital Medicine, San Francisco Veterans Affairs Medical Center, San Francisco, CA USA; 3 VA Center for Innovation to Implementation (Ci2i), Menlo Park, CA USA; 4 The Center for Access and Delivery Research and Evaluation (CADRE, Iowa City VA Healthcare System, Iowa City, IA USA; 5 Department of Internal Medicine, University of Iowa Carver College of Medicine, Iowa City, IA USA; 6 Division of Primary Care and Population Health, Stanford University School of Medicine, Stanford, CA USA

**Keywords:** telemedicine, virtual care, health services access, veterans health administration

## Abstract

**Introduction:**

The Veterans Health Administration (VHA) distributes video-enabled tablets to individuals with barriers to accessing care. Data suggests that many tablets are under-used. We surveyed Veterans who received a tablet to identify barriers that are associated with lower use, and evaluated the impact of a telephone-based orientation call on reported barriers and future video use.

**Methods:**

We used a national survey to assess for the presence of 13 barriers to accessing video-based care, and then calculated the prevalence of the barriers stratified by video care utilization in the 6 months after survey administration. We used multivariable modeling to examine the association between each barrier and video-based care use and evaluated whether a telephone-based orientation modified this association.

**Results:**

The most prevalent patient-reported barriers to video-based care were not knowing how to schedule a visit, prior video care being rescheduled/canceled, and past problems using video care. Following adjustment, individuals who reported vision or hearing difficulties and those who reported that video care does not provide high-quality care had a 19% and 12% lower probability of future video care use, respectively. Individuals who reported no interest in video care, or did not know how to schedule a video care visit, had an 11% and 10% lower probability of being a video care user, respectively. A telephone-based orientation following device receipt did not improve the probability of being a video care user.

**Discussion:**

Barriers to engaging in virtual care persist despite access to video-enabled devices. Targeted interventions beyond telephone-based orientation are needed to facilitate adoption and engagement in video visits.

**Supplementary Information:**

The online version contains supplementary material available at 10.1007/s11606-023-08468-x.

## INTRODUCTION

The Veterans Health Administration (VA) is the largest integrated health care system in the USA, with approximately 9 million Veterans enrolled.^1^ With nearly one-quarter of Veterans living in rural or highly rural areas, maintaining safe and consistent access to care for these individuals is a high priority for the VA.^2^ In 2016, the VA’s Offices of Rural Health and Connected Care initiated a program to facilitate video visits through the distribution of tablets to Veterans with geographic, clinical, and social barriers to care.^3^ Four years later, in response to the COVID-19 pandemic, the VA further increased its distribution of tablets to maintain consistent and safe access to care during this time. Between 2020 and 2022, approximately145,000 Veterans received VA-issued video-enabled tablets.

While the VA’s tablet distribution program has helped overcome a primary barrier in accessing virtual care (i.e., lacking a digital device that can perform video-based communication), other secondary barriers to video visits remain. These barriers include, but are not limited to, low levels of digital health literacy, lack of understanding of benefits, lack of training, poor Wi-Fi, and difficulty scheduling telehealth visits, to name a few.^4^ A recent VA Office of Inspector General (OIG) report found that among individuals who received a VA-issued tablet in the first three quarters of 2021, only half (51%) had a video-based appointment.^5^ Others have found that as many as 58% of tablet recipients have at least one video-based visit within 6 months^6^, although rates are much lower (46%) among certain populations, such as those experiencing homelessness.^7^ Such findings suggest downstream barriers to using these devices continue to exist. To overcome some of these barriers, the VA has implemented a one-time orientation phone call in which the VA attempts to call each tablet recipient and provide a set-up and orientation primer. To date, it is unclear how this orientation call effects individuals’ experiences and tablet use.

As video-based care continues to expand both within the VA and in other health care systems, there is a pressing need to understand the factors that inhibit engagement and to identify effective interventions. We hypothesized that Veterans who reported barriers to accessing video-based care would have lower subsequent use of video-based care compared to those who do not report such barriers. To assess this, we analyzed data from a national survey of VA tablet recipients to examine (a) the most commonly reported barriers to accessing video-based care after receiving a video-enabled tablet, (b) the relationship between barriers and use of video-based care, and (c) the impact of a VA Help Desk orientation phone call on reported barriers and future engagement in video-based care.

## METHODS

### Survey

We analyzed data from a national survey of Veterans who received a VA-issued, video-enabled tablet (Apple iPad) and who had regular use of VA health care (defined as having at least two primary care or mental health care visits in the prior 12 months ending in August 2021). The survey was conducted between September 2021 and January 2022, with all individuals identified using data from the VA’s Corporate Data Warehouse (CDW). All eligible individuals with an email address received an initial email with the option to complete the survey online or to call-in. Those who did not have an email on file or did not respond were mailed a survey packet with a pre-paid return envelope or provided instructions on completing the survey via phone or opting out. Non-responders were sent another letter and were contacted by phone if needed. The survey included questions about patient sociodemographics and barriers that prevented Veterans’ use of the VA’s telemedicine service, called VA Video Connect.^8^ This survey was conducted as part of a non-research quality improvement project supported by VA’s Office of Connected Care and was exempt from Institutional Review Board approval.

### Patient Characteristics

Basic demographics (age, rurality) and clinical characteristics (number of chronic conditions, mental health and substance use disorder diagnoses) were obtained from the CDW. Gender, race, and other sociodemographic characteristics (education, annual household income, difficulty paying for basics, co-habitation with others, and social support) were obtained through the survey. Broadband access was determined using Microsoft Airband Initiative data.^9^ Using electronic health data drawn from the CDW, survey respondents were categorized into two groups, i.e., “video users” and “video non-users,” based on their utilization of video-based primary care, subspecialty care, mental health care, and physical rehabilitation care in the 6 months after survey administration.

The survey also assessed for the presence of 13 barriers to accessing video-based care: problems using video care in the past, prior video care rescheduled/canceled, do not know how to schedule video care, no family/friends/caregivers to help with video care, belief that video care does not provide high-quality care, nervous about learning how to use video care, no cellular or internet connection, cannot focus enough to use video care, no interest in video care, unaware that I can see providers by video, vision or hearing difficulties preventing video care, lack of private space for video care, and physical difficulties preventing video care. These questions were created by the research team with input from leadership from the VA’s Office of Connected Care based on their knowledge studying and implementing virtual care within the VA (Supplement Table 1).

In addition to these variables, the survey asked if the patient had any interaction with the VA Help Desk service—a telephone-based orientation program the VA offers all tablet recipients. In this service, an IT specialist calls the patient and helps them sign onto the device, performs a video test call, and answers any further questions about how to operate the VA-issued tablets. Call scripts outlining these conversations are available in the supplement.

### Study Population

**Figure 1:**
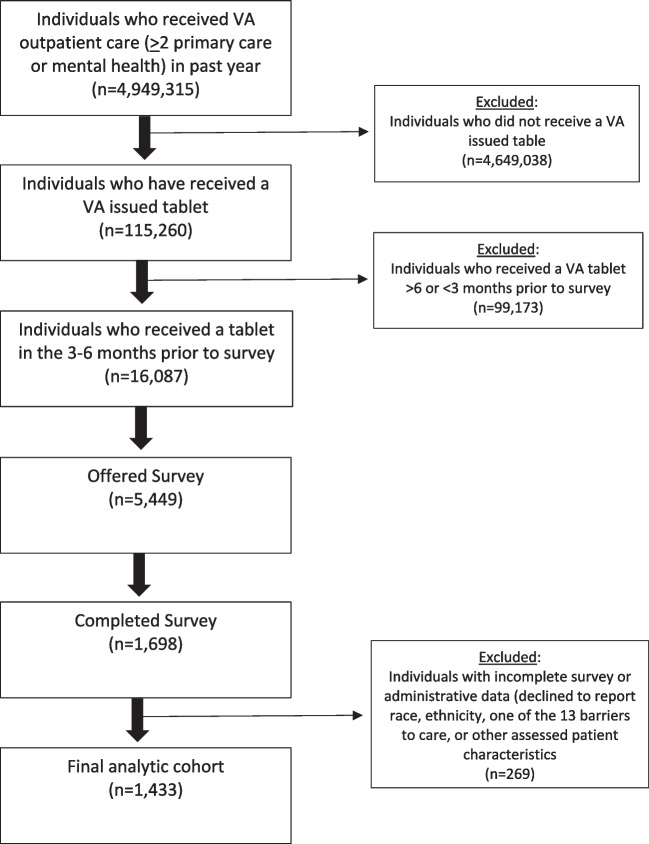
Consort diagram of analytic cohort.

Analysis focused on individuals with complete survey data and excluded those with incomplete survey or administrative data (declined to report race, ethnicity, one of the 13 barriers to care, or other assessed patient characteristics). A total of 269 individuals were excluded from final analysis. See flow chart (Fig. 1) for details.

### Outcomes

Our primary outcome was the use of video visits in the 6 months following survey administration. Six months was selected for the follow-up period as tablets are distributed based on a current need for VA health care. We included outpatient primary care, subspecialty, mental health, and rehabilitation visits that occurred via video. We identified these video-based outpatient encounters using VA’s Managerial Cost Accounting Stop Codes, which characterize VA outpatient clinic encounters and modality of care (e.g., in-person, video), and identify responsible work groups for documented clinical services as previously defined.^10^

### Statistical Analysis

We calculated the prevalence of the 13 barriers to video visit use among survey respondents, stratifying by video care utilization in the 6 months following survey administration. We examined the co-occurrence of each barrier (i.e., joint distribution) and report the proportion of individuals reporting each barrier among individuals reporting another barrier.

We used a generalized linear model with a Poisson distribution to examine the association between the 13 potential barriers to using video care and use of video-based care in the following 6 months, adjusting for characteristics that are associated with video care engagement (e.g., age, gender, race/ethnicity, urbanicity, education, annual household income, area broad band usage, difficulty paying for basic expenses, living alone, social support, number of chronic conditions, and presence of a mental health diagnosis). Additionally, we adjusted for each of the 13 barriers to report their independent association with subsequent video care use. We report average marginal effects (AME), which reflect the change in the probability of video care use by reported barrier.

To evaluate whether the VA Help Desk orientation phone call modified the relationship between barriers and video care use, we repeated the above analyses stratified based on VA Help Desk usage. All models are reported with 95% confidence intervals and standard error estimation accounted for the complex sample design of the survey. We conducted all statistical analyses using Stata 17.0 (StataCorp, LLC).

## RESULTS

Among 16,087individuals who received a VA-issued tablet during the sampling time frame, 5449 were invited to complete the survey and 1698 (31.2%) completed it (1433 of whom were included in analyses for this paper). Among all respondents (*n*=1433), almost half (44.6%) were >65 years old, most were male (84.4%) and reported being white (54.6%). Most respondents reported living in an urban environment (70.3%) and having poor broadband access (69.4%). One-third reported at least some college or 2-year degree (67.7%). Although most individuals reported living with other individuals (59.8%), approximately half stated they lacked social support (51.5%). Two-thirds (62.4%) of individuals who received a tablet reported receiving a VA Help Desk orientation phone call (Table 1). Three patient characteristics varied between survey respondents and non-respondents, namely gender (female respondents: 13.9 vs. 11.0%; *p*<0.003), use of VVC prior to survey (respondent: 76.6% vs. 64%; *p*<0.01), and use of VVC after the survey (respondents: 60.6% vs. 42.4%;* p*<0.01) (Supplement Table 2).

**Table 1:** Demographic, Clinical, and Social Characteristics of Study Population

	Total *N*=1433		Video care use in 6 months post survey			
			Non-users *N*=624		Users *N*=809	
	*N*	%	*N*	%	*N*	%
Age						
18–44	180	(12.6%)	44	(7.1%)	136	(16.8%)
45–64	614	(42.8%)	228	(36.5%)	386	(47.7%)
65+	639	(44.6%)	352	(56.4%)	287	(35.5%)
Gender						
Male	1210	(84.4%)	543	(87.0%)	667	(82.4%)
Female	209	(14.6%)	74	(11.9%)	135	(16.7%)
Non-binary gender	14	(1.0%)	7	(1.1%)	7	(0.9%)
Race						
Black/African American	401	(28.0%)	169	(27.1%)	232	(28.7%)
American Indian/Alaska Native	22	(1.5%)	13	(2.1%)	9	(1.1%)
Native Hawaiian/Pacific Islander/Asian	17	(1.2%)	5	(0.8%)	12	(1.5%)
Hispanic or Latino	111	(7.7%)	38	(6.1%)	73	(9.0%)
White	784	(54.7%)	361	(57.9%)	423	(52.3%)
Other	32	(2.2%)	14	(2.2%)	18	(2.2%)
More than one race	66	(4.6%)	24	(3.8%)	42	(5.2%)
Urban vs. rural/highly rural						
Urban	1009	(70.4%)	420	(67.3%)	589	(72.8%)
Rural/highly rural	424	(29.6%)	204	(32.7%)	220	(27.2%)
Education						
Did not complete high school	56	(3.9%)	31	(5.0%)	25	(3.1%)
High school grad or GED	402	(28.1%)	194	(31.1%)	208	(25.7%)
Some college or 2-year degree	671	(46.8%)	277	(44.4%)	394	(48.7%)
4-year college grad or more	304	(21.2%)	122	(19.6%)	182	(22.5%)
Annual household income						
Less than $25k	599	(41.8%)	280	(44.9%)	319	(39.4%)
$25,501–50,000	555	(38.7%)	236	(37.8%)	319	(39.4%)
More than $50,000	279	(19.5%)	108	(17.3%)	171	(21.1%)
Broadband access						
0–80%	990	(69.1%)	457	(73.2%)	533	(65.9%)
>80–100%	415	(29.0%)	154	(24.7%)	261	(32.3%)
No data	28	(2.0%)	13	(2.1%)	15	(1.9%)
Difficulty paying for basics						
Not difficult at all	499	(34.8%)	226	(36.2%)	273	(33.7%)
Somewhat difficult	650	(45.4%)	279	(44.7%)	371	(45.9%)
Very difficult	284	(19.8%)	119	(19.1%)	165	(20.4%)
Live with others or alone						
Alone	578	(40.3%)	267	(42.8%)	311	(38.4%)
With others	855	(59.7%)	357	(57.2%)	498	(61.6%)
Lacks social support						
Has social support	694	(48.4%)	307	(49.2%)	387	(47.8%)
Lacks social support	739	(51.6%)	317	(50.8%)	422	(52.2%)
Number of chronic conditions						
0	204	(14.2%)	75	(12.0%)	129	(15.9%)
1	186	(13.0%)	71	(11.4%)	115	(14.2%)
2–3	494	(34.5%)	203	(32.5%)	291	(36.0%)
4–5	324	(22.6%)	150	(24.0%)	174	(21.5%)
6+	225	(15.7%)	125	(20.0%)	100	(12.4%)
Any mental health condition	1120	(78.2%)	417	(66.8%)	703	(86.9%)
Any substance use disorder	572	(39.9%)	211	(33.8%)	361	(44.6%)
VA Help Desk usage	893	(62.3%)	385	(61.7%)	508	(62.8%)

### Prevalence of Sociodemographic Characteristics and Digital Barriers

Approximately 56.5% of individuals had a subsequent video encounter within 6 months after survey administration (i.e., video care users). Compared to video care users, non-users were older (>65 years; 56.4% vs. 35.5%), more likely to be male (87.0% vs. 82.4%), and White (58.0% vs. 52.0%). Non-users were also more likely to report qualities associated with decreased digital access, such as living in rural or highly rural areas (32.7% vs. 27.3%), having lower educational status (high school graduate or GED: 31.1% vs. 25.7 %), low household income (<$25k annually: 44.9% vs. 39.4%), and inadequate broadband access (% of households in the respective county that have broadband speed [25/3 Mbps]; 0–80%: 73.4% vs. 66.3%). Additionally, non-users had higher numbers of chronic conditions, but fewer mental health conditions or histories of substance use disorders. Similar proportions of video care users and non-users reported receiving a call from the VA Help Desk (61.7% vs. 62.8%) (Table 1). A distribution of Veteran characteristics and the reported barriers was also calculated (Supplement Table 4).

**Figure 2:**
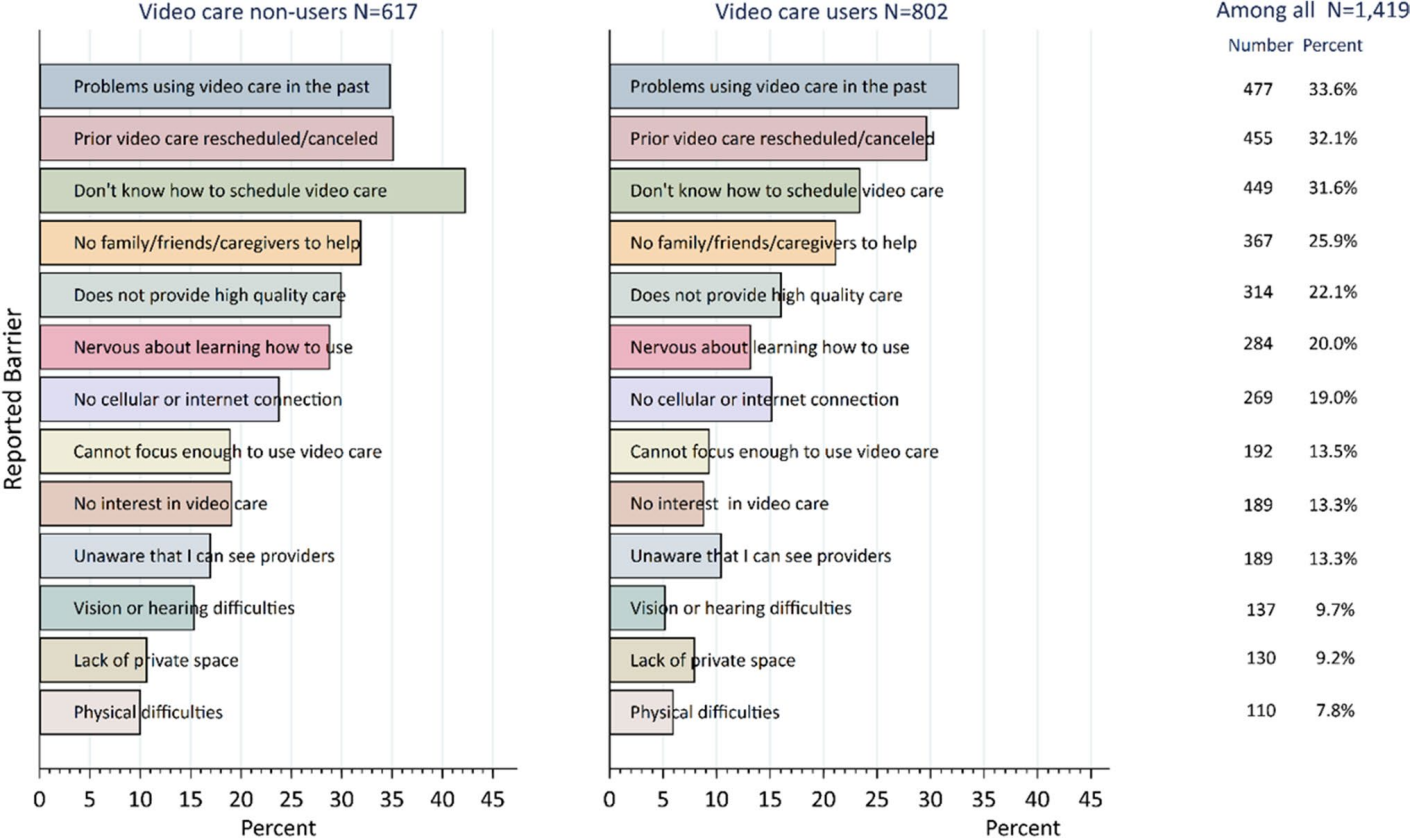
Prevalence of survey reported barriers among 1433 survey respondents by no and any video care use in the 6 months after survey administration.

On average, respondents reported 2.5 barriers, with 69% stating they had at least one barrier (Supplement Figure 1 and Table 3). Among video care non-users, the most prevalent reported barriers were as follows: not knowing how to schedule video care (42.5%), prior video care being rescheduled or canceled (35.3%), and having problems using video care in the past (34.8%). While Veterans who used video care had reported similar barriers to non-users in the survey (problems using video care in the past [32.8%], prior video care being rescheduled [29.8%], and not knowing how to schedule video care [23.2%]), the overall prevalence of these reported barriers, and all other barriers, was lower (Fig. 2).

In assessing co-occurrence of barriers, we found that the presence of one barrier was always associated with a higher prevalence of another barrier (Supplement Table 2).

### Impact of Barriers on Video Care Utilization

**Figure 3:**
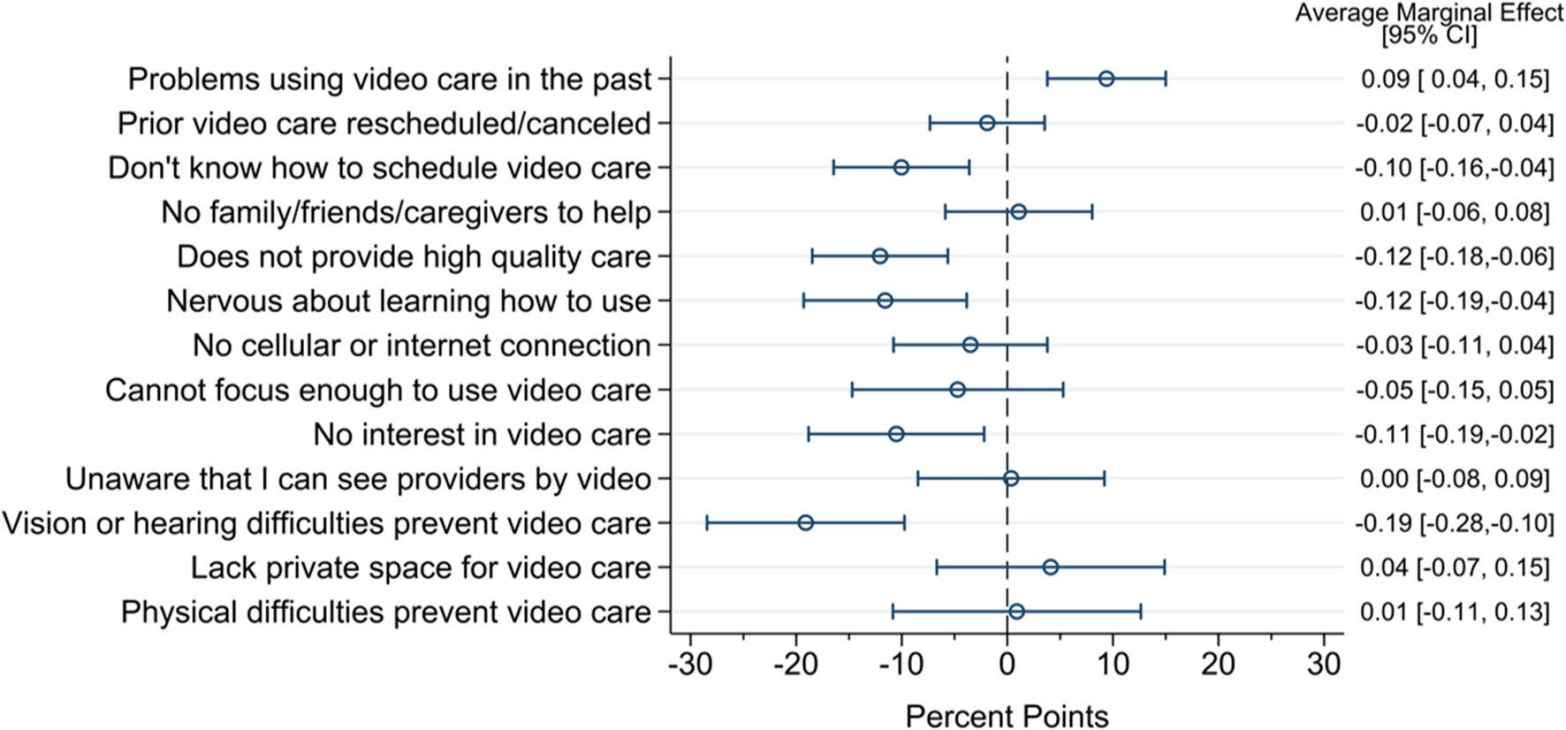
Change in the probability of being a video care user by reported barrier.

In adjusted models assessing the relationship between barriers and the probability of being a video care user, we found that probability of video care use was significantly lower among individuals who report the following barriers: vision or hearing difficulties (AME −0.19 [95% CI −0.28, −0.10]), video care does not provide high-quality care (AME −0.12 [95% CI −0.18, −0.05]), nervousness about learning how to use the device (AME −0.12 [95% CI −0.19, −0.04]), no interest in video care (AME −0.11 [95% CI −0.19, −0.02]), and lack of knowledge about how to schedule a video care visit (AME −0.10 [95% CI −0.17, −0.04]). Interestingly, those who reported having problems in the past were 9% more likely to have a video visit in the subsequent 6 months (AME 0.09 [95% CI 0.03, 0.15]). There were no statistically significant relationships between other reported barriers and video use (Fig. 3).

### Impact of VA Help Desk on Video Care Utilization

**Figure 4:**
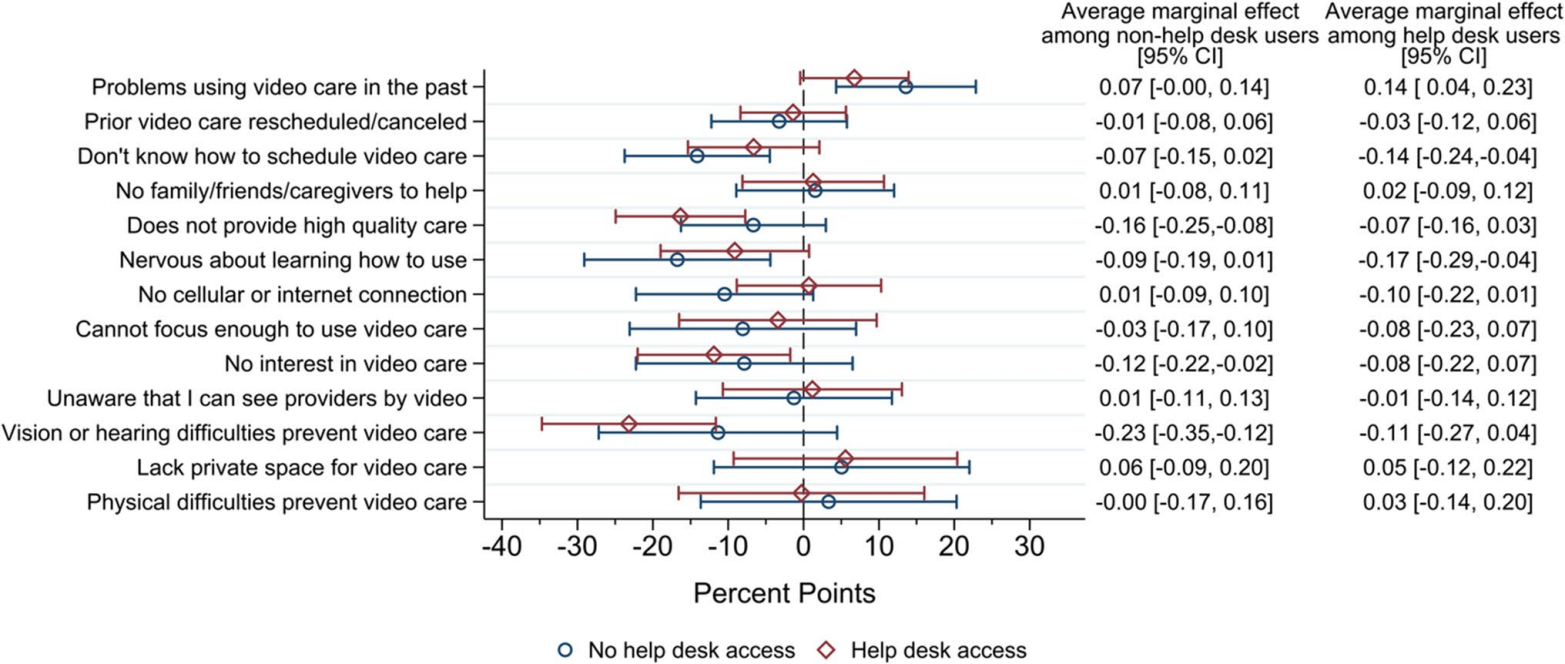
Change in the probability of being a video care user by reported barrier: stratified by history of Help Desk use.

In stratified analysis, we found that survey respondents who reported receiving a VA Help Desk orientation call did not have an increased probability of having a video visit in the subsequent 6 months, and results were consistent across groups that reported specific barriers (Fig. 4).

## DISCUSSION

In this national survey of VA tablet recipients, we found that despite having access to a video-enabled tablet, many individuals continued to report barriers to engaging in video-based care. Systems issues (e.g., problems with video care in the past and difficulties scheduling video care) were the most prevalent barriers and were reported at similar rates among those who subsequently had and did not have a video visit. Individuals who reported vision or hearing difficulties, uncertainty around the quality of video-based care, or nervousness about using video-based care were least likely to subsequently use video care. None of the relationships between barriers and video care use appeared to be modified by a patient’s interaction with the VA Help Desk. These findings are informative for several reasons.

First, the prevalence of barriers highlights that having a video-enabled device is necessary but not sufficient for video visit engagement. Secondary barriers, such as not knowing how to schedule a video visit or problems using video care, also influence the use of video-based services. Similar findings have been found in other health care environments, populations, and systems.^11–13^ For example, a recent review found secondary barriers, such as negative perceptions of virtual care, inadequate clinical workflows, and insufficient virtual care training and education, were common obstacles associated with non-use of telehealth services in primary care settings.^14^ Additionally, a recent study by Loizos et al. found that even when a group of homebound adults were provided free video-enabled tablets, only one-third used them.^15^ The authors determined that lack of comfort and digital skills were major contributors to lack of use. Altogether, our findings, in conjunction with these other studies, indicate that offering access to a video-enabled device is not an adequate intervention without addressing users’ ability and comfort in using the device.

Second, our findings describe specific barriers that health care systems could focus on as they attempt to increase engagement in virtual care. For example, we found individuals with functional disabilities (e.g., hearing or vision impairment) may be a specific population for whom greater resources, education, and support could be valuable. Recent studies have shown that individuals with communication and hearing barriers have lower treatment adherence, increased medical costs, and hospital readmissions.^16, 17^ If health care systems hope to achieve equitable access and use of telehealth services, identifying and engaging those with functional limitations to engaging in such care will be key.

Third, our work highlights the importance of considering patients’ trust in virtual care interactions and their concerns regarding the quality of care delivered via telehealth modalities. While studies find that patients appreciate the conveniences of virtual care (e.g., decreased travel times and costs, faster access to providers),^11, 18, 19^ some have raised concerns about implications for building and maintaining patient-provider trust during these interactions.^18^ In a recent study that measured patient trust in telemedicine services, Velsen et al. found that, while trust in the health care professional was rarely an issue, distrust in the technology and the telehealth platform was a more salient concern for many patients.^20^ As telehealth becomes more ubiquitous, health systems will need to identify technologies and processes that patients perceive as trustworthy.^21^ In addition, more research is needed to identify how telehealth can be leveraged to provide high-quality care, and when in-person services should be prioritized.

Finally, this work highlights the need for digital literacy training among digitally vulnerable populations. While the VA offers an orientation telephone call to all new tablet recipients (e.g., VA Help Desk), our findings suggest this one-time intervention may not be sufficient. Recent work by Castilla et al. found that offering older individuals multiple digital literacy training sessions enabled them to independently use a digital interface, and that continued use of the interface improved user perceptions and comfort with the digital tool.^22^ If health care systems hope to engage their most digitally vulnerable populations (e.g., older patients with multiple comorbid conditions) in virtual-based care, resources and more in-depth digital training may be required.

While this study did not explore specific ways to address assessed barriers, prior work suggests some novel ways to overcome secondary barriers to digital use. These include deploying community health workers or digital trainers to digitally vulnerable patients or populations^23^, using social networks to improve digital literacy,^22^ and providing digital training during hospitalization or other moments when patients are a “captured audience.”^24^ We note that before such interventions are pursued, health systems will need better and more robust systematic data collection on patient’s digital skills, abilities, and comfort. Without such information, focused and meaningful interventions will be difficult to implement. While such interventions will require upfront investments, time, and resources, studies suggest such programs could lead to downstream cost savings.^25^ Finally, health care systems could advocate for more human-centered design approaches, for example, creating or adopting technologies that do not require as much training or effort to use.

Our study has limitations. First, this study was conducted among tablet recipients who receive their care in the VA health care system; thus, findings may not be generalizable beyond this population and setting. Second, our assessment of barriers was limited to the questions that were included in the survey. Thus, we may not be capturing all potential barriers to accessing video-based care. Third, we relied on participants’ survey responses to elicit whether they engaged with the VA Help Desk and were unable to verify or assess the quality of these Help Desk interactions. Fourth, our assessment focused on video-based care and did not examine telephone-based telehealth, which is the predominant form of virtual care in the VA. We made this decision as video visits are the VA’s preferred modality for virtual visits that are substituting for in-person clinical encounters. Finally, response biases and exclusion of individuals who did not complete all relevant survey questions may influence generalizability of findings.

In conclusion, we found that barriers to engaging in video visits persist even once a person is given a video-enabled device, and those barriers reduce the likelihood of a person engaging in video-based care. A single telephone-based orientation after device receipt is insufficient to overcome these barriers. As the VA and other health care systems continue to increase their use of telehealth services, these findings can inform the development of policies, interventions, and training to address access barriers and increase adoption and use of virtual care technologies.

### Supplementary Information

Below is the link to the electronic supplementary material.

Supplementary file1 (XLSX 16 KB)

Supplementary file2 (DOCX 45 KB)

## Data Availability

Due to US Department of Veterans Affairs (VA) regulations and our ethics agreements, the analytic data sets used for this study are not permitted to leave the VA firewall without a Data Use Agreement. This limitation is consistent with other studies based on VA data. However, VA data are made freely available to researchers with an approved VA study protocol. For more information, please visit https://www.virec.research.va.gov or contact the VA Information Resource Center at VIReC@va.gov.

## Declarations:

### Disclaimer:

Views expressed are those of the authors and the contents do not represent the views of the US Department of Veterans Affairs or the United States Government.
