# Supplementary material for: Overcoming Obstacles: Barriers to Virtual Care Use Among Video-Enabled Tablet Recipients in the Veterans Health Administration
Source: J Gen Intern Med. 2023 Nov 1;39(4):549–56. doi: 10.1007/s11606-023-08468-x (PMC10973323; doi:10.1007/s11606-023-08468-x)
Supplement: Supplementary file 2 — Supplementary file2 (DOCX 45 KB) [file 11606_2023_8468_MOESM2_ESM.docx]

**SUPPLEMENTAL MATERIALS**

**Table 1.** Barriers Questions from the Survey

**Do any of the following prevent you from have a VA video visit?**

|  | **Yes** | **No** |
| --- | --- | --- |
| I have no interest |  |  |
| I am unaware I can see providers by video |  |  |
| I do not have cellular or internet connection |  |  |
| I do not know how to schedule video visits |  |  |
| I do not have family, friends, or caregivers to help me |  |  |
| I cannot focus enough to use video visits |  |  |
| I feel nervous about learning how to use video visits |  |  |
| My vision or hearing prevents me from using video visits |  |  |
| Physical mobility prevents me from using video visits |  |  |
| I do not have a private space for video visits |  |  |
| Video visits do not provide high quality of care for my health needs |  |  |
| I had problems using video visits in the past |  |  |
| I was scheduled for a video visit, but it was cancelled or rescheduled |  |  |

**Table 2.** Respondent vs. Non-Respondent Patient Characteristics

|  |  | **Cohort 1: Tablet Recipients** | | | |
| --- | --- | --- | --- | --- | --- |
|  |  | **Nonresponse** | **Respondent** | **p** | **SMD** |
| n |  | 3752 | 1697 |  |  |
| **Age (Mean, SD)** |  | 63.12 (13.71) | 63.56 (13.27) | 0.275 | 0.032 |
|  | 18-44 | 475 (12.7) | 181 (10.7) | 0.109 | 0.063 |
|  | 45 - 64 | 1384 (36.9) | 635 (37.4) |  |  |
|  | 65+ | 1893 (50.5) | 881 (51.9) |  |  |
| **Gender** | F | 413 (11.0) | 236 (13.9) | 0.003 | 0.088 |
|  | M | 3339 (89.0) | 1461 (86.1) |  |  |
| **Race and Ethnicity** | Black or African American | 1151 (30.7) | 473 (27.9) | 0.242 | 0.069 |
|  | Hispanic or Latino | 205 ( 5.5) | 104 ( 6.1) |  |  |
|  | Other | 184 ( 4.9) | 78 ( 4.6) |  |  |
|  | Unknown/Missing | 147 ( 3.9) | 72 ( 4.2) |  |  |
|  | White | 2065 (55.0) | 970 (57.2) |  |  |
| **VA Enrollment Priority** | 1. Priority 7-8 | 279 ( 7.4) | 111 ( 6.5) | 0.332 | 0.065 |
|  | 2. Priority 5 (low income) | 983 (26.2) | 433 (25.5) |  |  |
|  | 3. Priority 1 & 4 (high disability) | 1907 (50.8) | 861 (50.7) |  |  |
|  | 4. Priority 2-3-6 (low/moderate disability) | 581 (15.5) | 292 (17.2) |  |  |
|  | MISSING | 2 ( 0.1) | 0 ( 0.0) |  |  |
| **History of Unstable Housing** |  | 909 (24.2) | 369 (21.7) | 0.049 | 0.059 |
| **Urban/Rural Status** | Missing | 1 ( 0.0) | 0 ( 0.0) | 0.719 | 0.027 |
|  | Rural/Highly Rural | 1122 (29.9) | 518 (30.5) |  |  |
|  | Urban | 2629 (70.1) | 1179 (69.5) |  |  |
| **Broadband Usage** | >80-100% | 1115 (29.7) | 495 (29.2) | 0.431 | 0.039 |
|  | 0-80% | 2547 (67.9) | 1170 (68.9) |  |  |
|  | No Data | 90 ( 2.4) | 32 ( 1.9) |  |  |
| **Number Chronic Conditions** (Mean, SD) |  | 7.51 (3.82) | 7.45 (3.66) | 0.573 | 0.017 |
| **Any mental health condition** |  | 3001 (80.0) | 1330 (78.4) | 0.185 | 0.04 |
| **Any VVC visit 1 year prior to sampling (%)** |  | 2405 (64.1) | 1300 (76.6) | <0.001 | 0.276 |
| **Any VVC visit 6 months after sampling (%)** |  | 1589 (42.4) | 1028 (60.6) | <0.001 | 0.371 |

**Figure 1**. Distribution of number of barriers reported among survey respondents; n=1,433

**
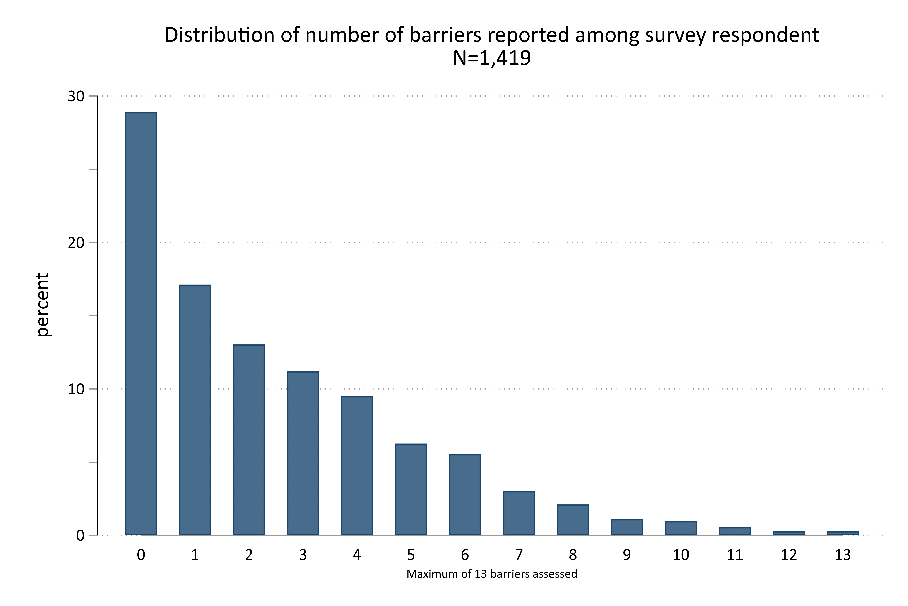
**

**Table 1**. Barrier Count, Frequency and Percent among survey respondents; n=1,433

| **Barrier Count** | **Frequency** | **Percent** |
| --- | --- | --- |
| 0 | 410 | 28.9 |
| 1 | 243 | 17.1 |
| 2 | 185 | 13.0 |
| 3 | 159 | 11.2 |
| 4 | 135 | 9.5 |
| 5 | 89 | 6.3 |
| 6 | 79 | 5.6 |
| 7 | 43 | 3.0 |
| 8 | 30 | 2.1 |
| 9 | 16 | 1.1 |
| 10 | 14 | 0.99 |
| 11 | 8 | 0.56 |
| 12 | 4 | 0.28 |
| 13 | 4 | 0.28 |

**Table 3.** Barrier Co-Occurrence Rates

|  | N | Problems using in past | Prior video care rescheduled/ canceled | Don't know how to schedule | No family/ friends/ caregiver to help | Does not provide high quality care | Nervous about learning how to use | No cellular or internet connection | Cannot focus enough to use video care | No interest in video care | Unaware that I can see providers | Vision or hearing difficulties | Lack of private space | Physical difficulties |
| --- | --- | --- | --- | --- | --- | --- | --- | --- | --- | --- | --- | --- | --- | --- |
|  |  |  |  |  |  |  |  |  |  |  |  |  |  |  |
|  |  |  |  |  |  |  |  |  |  |  |  |  |  |  |
| All Persons | 1433 | 33 | 32 | 31 | 25 | 22 | 20 | 19 | 13 | 13 | 13 | 10 | 9 | 8 |
| Problems using video care in the past | 477 | - | 54 | 48 | 39 | 38 | 37 | 26 | 26 | 21 | 20 | 17 | 15 | 15 |
| Prior video care rescheduled/canceled | 455 | 57 | - | 43 | 35 | 36 | 28 | 26 | 23 | 17 | 20 | 17 | 15 | 13 |
| Don't know how to schedule video care | 449 | 51 | 43 | - | 51 | 38 | 43 | 33 | 29 | 24 | 27 | 17 | 16 | 15 |
| No family/friends/caregivers to help | 367 | 51 | 43 | 63 | - | 38 | 38 | 39 | 32 | 24 | 25 | 19 | 19 | 15 |
| Does not provide high quality care | 314 | 57 | 52 | 55 | 44 | - | 39 | 28 | 34 | 30 | 26 | 21 | 20 | 19 |
| Nervous about learning how to use | 284 | 61 | 45 | 67 | 49 | 43 | - | 31 | 39 | 27 | 23 | 26 | 18 | 18 |
| No cellular or internet connection | 269 | 47 | 44 | 55 | 53 | 33 | 33 | - | 32 | 28 | 33 | 19 | 23 | 16 |
| Cannot focus enough to use video care | 192 | 65 | 54 | 68 | 61 | 56 | 58 | 45 | - | 38 | 34 | 38 | 32 | 30 |
| No interest in video care | 189 | 53 | 40 | 57 | 46 | 49 | 41 | 40 | 38 | - | 35 | 23 | 25 | 19 |
| Unaware that I can see providers by video | 189 | 50 | 47 | 63 | 49 | 44 | 34 | 47 | 34 | 35 | - | 21 | 21 | 18 |
| Vision or hearing difficulties prevent video care | 137 | 59 | 57 | 56 | 52 | 48 | 55 | 37 | 53 | 32 | 29 | - | 24 | 37 |
| Lack private space for video care | 130 | 56 | 52 | 56 | 53 | 49 | 39 | 47 | 48 | 36 | 31 | 25 | - | 26 |
| Physical difficulties prevent video care | 110 | 63 | 55 | 63 | 50 | 53 | 46 | 40 | 53 | 32 | 30 | 46 | 31 | - |
|  |  |  |  |  |  |  |  |  |  |  |  |  |  |  |
| Twice the prevalence among all persons |  |  |  |  |  |  |  |  |  |  |  |  |  |  |
| Three times the prevalence among all persons |  |  |  |  |  |  |  |  |  |  |  |  |  |  |
|  |  |  |  |  |  |  |  |  |  |  |  |  |  |  |

Numbers in the top row represent the percentage of survey respondents who reported the barrier in each column. In the remaining rows, the number in each cell represents the percentage of individuals with the row barrier who also report the column barrier.

**Connected Device Support (CDS) call script used to call tablet recipients**

**1^st^ Call Attempt:**

“Hello, this is [your first name] of the {VA Office of Connected Care Help Desk}.”

Am I speaking with [name of Veteran]?”

“{First and foremost [salutation, name],} **I would like to thank you for your service.**”

“I am calling in regard to your recently delivered Patient Video Tablet.”

“Have you received your iPad?”

1. If **Yes**, proceed with set up process
2. If **No**, If leaving a VM please state name, reason you are calling, let the Veteran know to plug in his device and charge it, let them know you will call them back in two days, and leave the OCCHD phone number if they need immediate assistance in setting up their iPad.

**Yes:** “Is now a good time for me to help you setup your device and get you ready for your first video appointment with your VA Provider?”

**Training and Talking Points**

- iPad Overview
  - iPad Button locations
  - Creating a Passcode (6-digit numeric)
    - Optional – Enabling Touch ID
- Testing the VA Issued iPad
  - {insert icon} VVC Test
    - Connection issues?
      - Verify Number of Bars, top left
      - Run {insert icon} Speed test – Verify minimum Video requirements are met (0.128Mbps down/up) referencing the included in shipment iPad Mini-Speedtest_TestVMR_v3.1.pdf document
- Setting up Email / Calendar for Scheduled Appointments
  - Always offer to set up email on Veterans iPad
  - Check and see if the Veteran has **Group** appointments. Group appointments will need email set up. If Veteran does not have an email, offer to set it up for them using a generic email provider, (Gmail).
- Scheduling a Video Visit
  - Day before visit – (iPad turned ON and left to charge overnight).
  - Day of visit – (iPad at least 75% or plugged in for appointment, enter VVC at least 5 mins before appointment)
- Entering Your VVC Appointment – Click the {insert icon} VVC icon
- Locating and Installing Apps – {insert icon} VA App Catalog

**End of Live call**

“Do you have any questions regarding the device I can assist you with?”

“The Office of Connected Care Help Desk is available to assist you with your new device 24 hours a day, 7 days a week for any questions or issues you may have.”

**OCCHD Help Desk**- 1866-651-3180

“Their phone number is listed on the back of the device and you can also find it on the Lock screen of the tablet as well.”

“**Thank you for your service**, and have a wonderful rest of your [day, week, evening, etc.]”

**3^rd^ Call Attempt:**

Hello, my name is _________ , I am calling from the VA Office of Connected Care to assist you with setting up your VA issued iPad. I have tried reaching you the last few days and have been unsuccessful. Please feel free to reach out to the OCCHD helpdesk at 1-866-651-3180 for further assistance. Thank you for your service and have a great day.

**Optional 4^th^ Attempt Calls- When time is allowable based off workload.**

If successful, change the special accommodations dropdown to MULTIPLE CONTACT ATTEMPTS (3+) NEEDED and then change the current disposition - Set Up Completed-After Third Attempt- Comments – Multiple contact.

**Notes:**

- Remember that your last call should be as good as your first call of the day.
- Take your time, do not interrupt Veteran when they are speaking.
- Be patient, don’t rush through the setup, allow enough time for the Veteran to ask questions.
- Make sure to remind Veteran to charge their devices the night before.
- If a Veteran uses profanity or starts yelling, try to redirect, ask them if this is not a good time and if they want to schedule for another day. Always be patient and polite. If a Veteran becomes belligerent over the phone, contact Edna immediately to join call.
- If you feel Veteran is becoming frustrated or inpatient with the process, offer them another time to set up.
- Remember the audience you are working with. We are setting up devices for a population that are elderly, have MH issues, low visual acuity, hearing difficulties or physical limitations. This is an older generation, be mindful of their lack of tech skills.
- Remember we are fortunate not to have a time limit on these calls. Take as much time needed to ensure the Veteran is comfortable as much as they can be with their iPad.

**Appointments- When time is allowable based off workload. Schedule appointments between 3-4**
